# Supplementary material for: Triggered contraction of self-assembled micron-scale DNA nanotube rings
Source: Nat Commun. 2024 Mar 14;15:2307. doi: 10.1038/s41467-024-46339-z (PMC10940629; doi:10.1038/s41467-024-46339-z)
Supplement: Supplementary file 1 — Supplementary Information [file 41467_2024_46339_MOESM1_ESM.pdf]

Supplementary Information: Triggered contraction of self-assembled micron-scale DNA nanotube rings

Maja Illig,[1,2†] Kevin Jahnke,[2,3†] Lukas P. Weise,[4†]
Marlene Scheffold,[2] Ulrike Mersdorf,[2] Hauke Drechsler,[5,6] Yixin Zhang,[5]
Stefan Diez,[5,7*] Jan Kierfeld,[4*] Kerstin Göpfrich[1,2*]

[1] Center for Molecular Biology of Heidelberg University (ZMBH),
Im Neuenheimer Feld 329, 69120 Heidelberg, Germany,
[2] Max Planck Institute for Medical Research, Biophysical Engineering Group,
Jahnstraße 29, 69120 Heidelberg, Germany,
[3] Harvard University, School of Engineering and Applied Sciences (SEAS),
Cambridge, MA, USA,
[4] TU Dortmund University, Department of Physics, Otto-Hahn-Str. 4, 44221 Dortmund, Germany,
[5] B CUBE - Center for Molecular Bioengineering and Cluster of Excellence Physics of Life,
Technische Universität Dresden, Tatzberg 41, 01307 Dresden, Germany,
[6] Tübingen University, Center for Plant Molecular Biology (ZMBP),
Auf der Morgenstelle 32, 72076 Tübingen, Germany,
[7] Max Planck Institute of Molecular Cell Biology and Genetics,
Pfotenhauerstrasse 108, 01307 Dresden, Germany
[*] Corresponding author e-mails: stefan.diez@tu-dresden.de, jan.kierfeld@tu-dortmund.de,
k.goepfrich@zmbh.uni-heidelberg.de
[†]These authors contributed equally.

Contents

Supplementary Figures **3**

Supplementary Figure 1: DNA bundles at high DNA nanotube concentrations 3

Supplementary Figure 2: Colocalization intensity of TAMRA-labeled synthetic peptides 4

Supplementary Figure 3: Colocalization of DNA nanotubes and starPEG-$(KA7)_4$ . . 5

Supplementary Figure 4: Bundle thickness analysis 6

Supplementary Figure 5: Colocalization intensity over time 7

Supplementary Figure 6: Yield of DNA rings 8

Supplementary Figure 7: Microscopic structure of DNA nanotube rings 9

Supplementary Figure 8: Dependence on $MgCl_2$ and DNA tile concentration 10

Supplementary Figure 9: DNA ring diameter is time-independent 11

Supplementary Figure 10: Analysis of the circularity of DNA rings 12

Supplementary Figure 11: DNA ring contraction with Methylcellulose 13

Supplementary Tables **14**

Supplementary Table 1: List of DNA sequences 14

Supplementary Notes **15**

Supplementary Note 1: Theory of bundle contraction 15

Supplementary Note 2: MD simulations 18

Supplementary Note 3: Ring contraction-driving parameters for physical system . . . 25

Supplementary Note 4: Dextran-induced depletion attraction 30

Supplementary Figures

Supplementary Figure 1: DNA bundles at high DNA nanotube concentrations

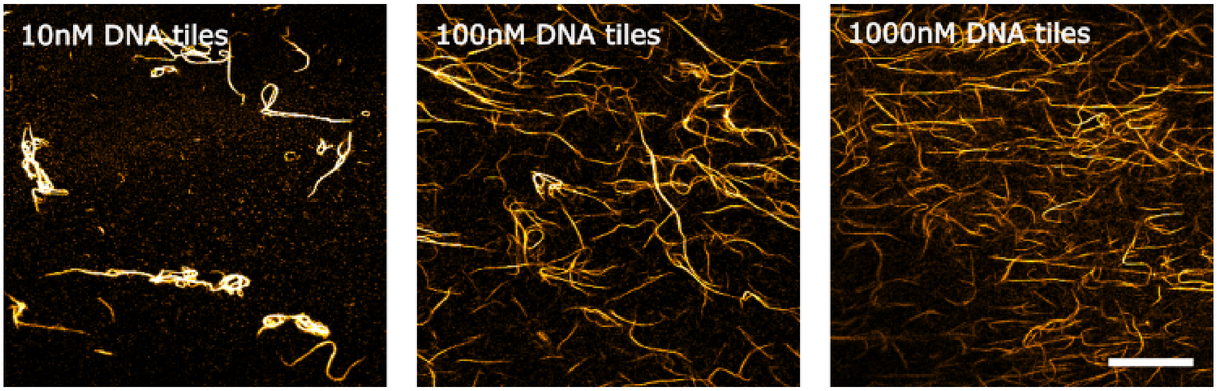

Supplementary Fig. 1: Increasing amounts of DNA nanotubes (formed from $10\,\mathrm{nM}$, $100\,\mathrm{nM}$, $1000\,\mathrm{nM}$ DNA tiles) were incubated at room temperature with $500\,\mathrm{nM}$ starPEG-(KA7)$_4$-TAMRA in 1x PBS and $10\,\mathrm{mM}$ $MgCl_2$. The DNA nanotube bundling correlates with the ratio of crosslinker concentration to DNA nanotube concentration. The laser settings were kept the same at all conditions. Scale bar: $30\,\mathrm{\mu m}$.

Supplementary Figure 2: Colocalization intensity of TAMRA-labeled synthetic peptides

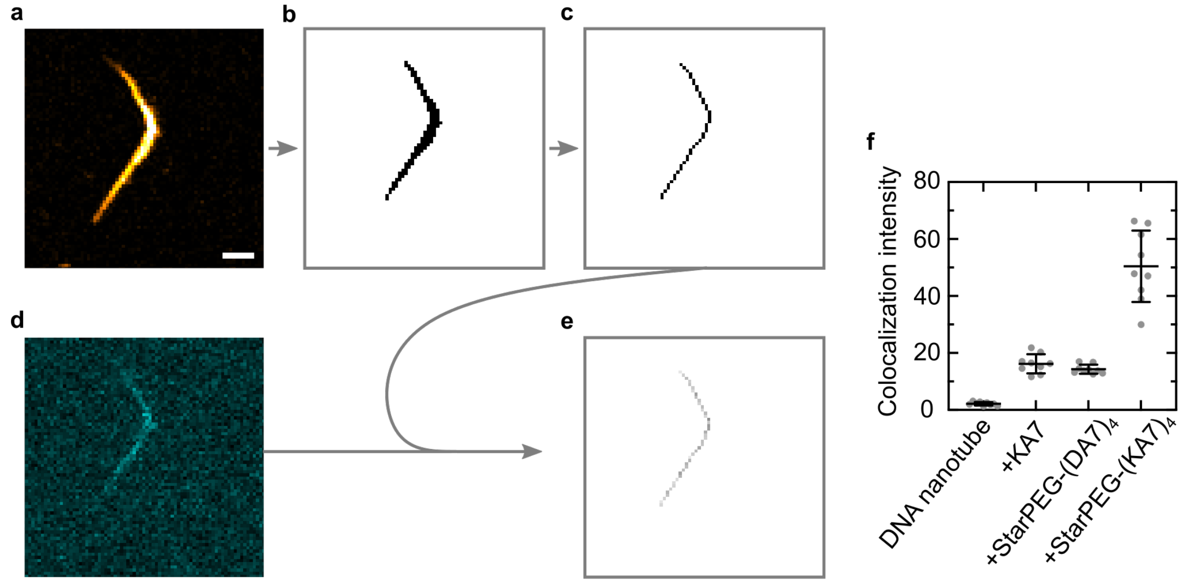

Supplementary Fig. 2: Mean pixel colocalization intensity c_p of TAMRA-labeled synthetic peptides. (**a**) Confocal image of $30\,\mathrm{nM}$ DNA nanotubes (yellow, Atto633, $\lambda_{ex} = 640\,\mathrm{nm}$) in presence of $500\,\mathrm{nM}$ starPEG-(KA7)$_4$ ($\lambda_{ex} = 561\,\mathrm{nm}$). Scale bar: 2 µm. The image was analyzed with ImageJ ((Fiji Is Just) ImageJ 2.3.0/1.5q; Java 1.8.0_322 64-bit) [9] and thresholded via the method Otsu (**b**) and the binary image was processed further with the Plugin Skeletonize (**c**) to reduce the location of the DNA nanotube bundle to the central line. Pixel values 0 and 1. (**d**) Confocal image of the same bundle in the starPEG-(KA7)$_4$ channel (cyan, $\lambda_{ex} = 561\,\mathrm{nm}$). Pixel values from 0 to 255. (**e**) starPEG-(KA7)$_4$ pixel intensity at the central line of the DNA nanotube bundle, calculated by the ImageCalculator function 'AND'. Pixel values from 0 to 255. (**f**) Colocalization intensity of TAMRA-labeled synthetic peptides ($\lambda_{ex} = 561\,\mathrm{nm}$) with the DNA nanotubes (mean ± standard deviation (SD), $n = 9$ analysed DNA nanotube bundles per condition). As expected, the colocalization intensity of starPEG-(KA7)$_4$ is the highest with 51.3 ± 12.5. The colocalization is significantly lower for KA7 and starPEG-(DA7)$_4$ (16.7 ± 3.3 and 14.1 ± 1.6, respectively).

Supplementary Figure 3: Colocalization of DNA nanotubes and starPEG-$(KA7)_4$

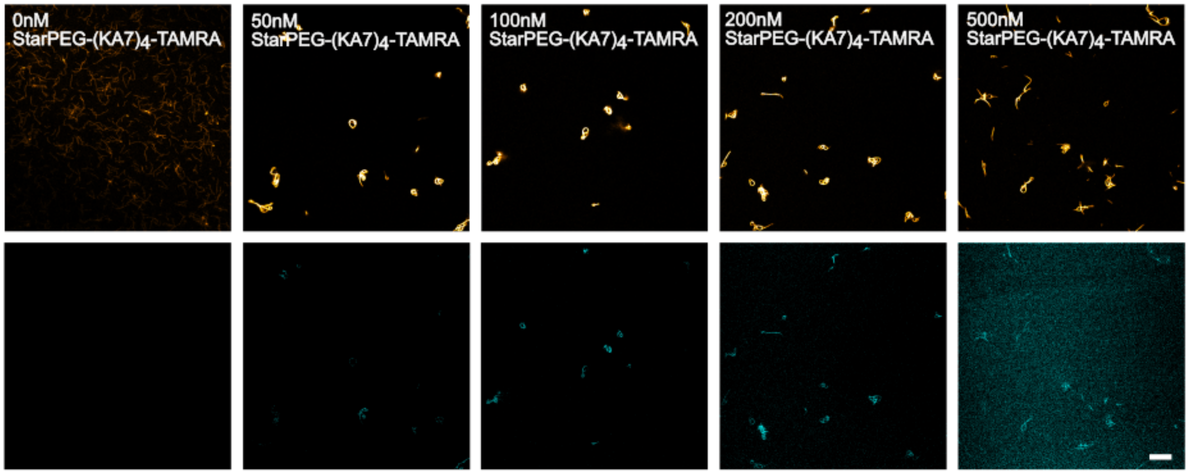

Supplementary Fig. 3: Colocalization of DNA nanotubes (top) and starPEG-$(KA7)_4$ (bottom). The mix contained starPEG-$(KA7)_4$-TAMRA (cyan) at varying concentrations (0, 50, 100, 200, 500 nM), DNA nanotubes (yellow) formed from 30 nM DNA tiles, 1× PBS and 10 mM $MgCl_2$. After incubation at room temperature for one hour the samples were imaged at the confocal laser scanning microscope with the same laser settings for all conditions. Scale bar: $10\,\mu m$.

Supplementary Figure 4: Transmission electron micrographs for bundle thickness analysis

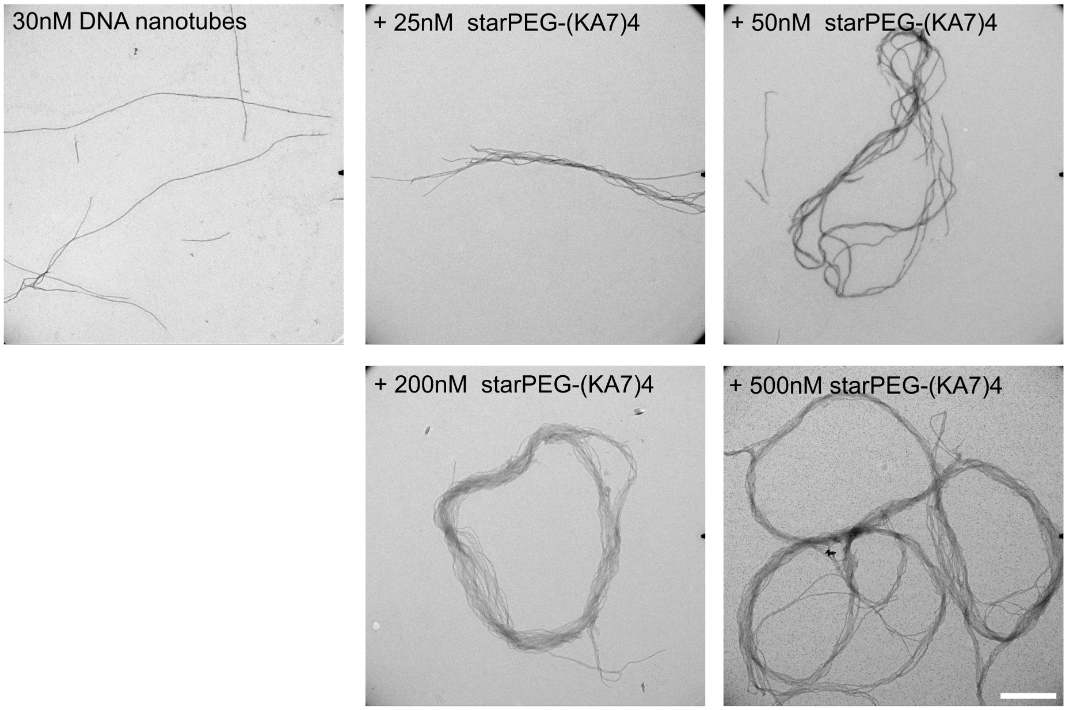

Supplementary Fig. 4: Transmission electron micrographs of DNA nanotube bundles. DNA nanotubes from $30\,\mathrm{nM}$ DNA tiles are bundled in the presence of 0, 25, 50, 200 and $500\,\mathrm{nM}$ starPEG-(KA7)$_4$ in 1× PBS and $10\,\mathrm{mM}$ $MgCl_2$. Bundles of different thicknesses were analyzed and the corresponding data is shown in Fig. 2**c**. Per condition the thicknesses were measured at ten different positions in each of a total of ten images. Scale bar: $500\,\mathrm{nm}$.

Supplementary Figure 5: Colocalization intensity over time

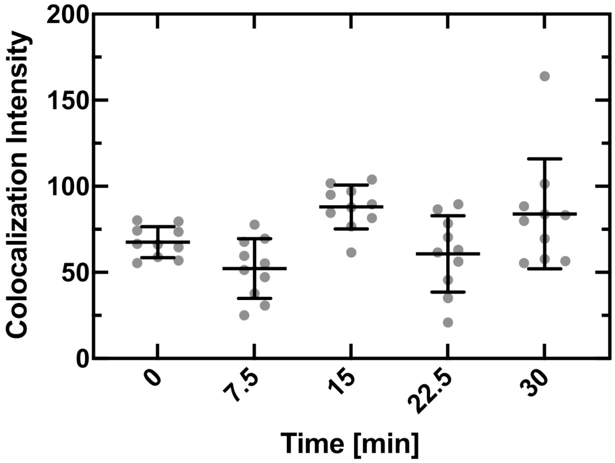

Supplementary Fig. 5: Colocalization intensity over time. DNA nanotubes formed from $30\,\mathrm{nM}$ DNA tiles were left to incubate with $500\,\mathrm{nM}$ starPEG-(KA7)$_4$-TAMRA at room temperature. The sample was imaged at $0, 7.5, 15, 22.5$ and $30\,\mathrm{min}$ after adding $500\,\mathrm{nM}$ starPEG-(KA7)$_4$-TAMRA. The laser settings were kept the same at all time points. For each condition 10 overview images were analyzed (Mean$\pm$SD).

Supplementary Figure 6: Yield of DNA rings

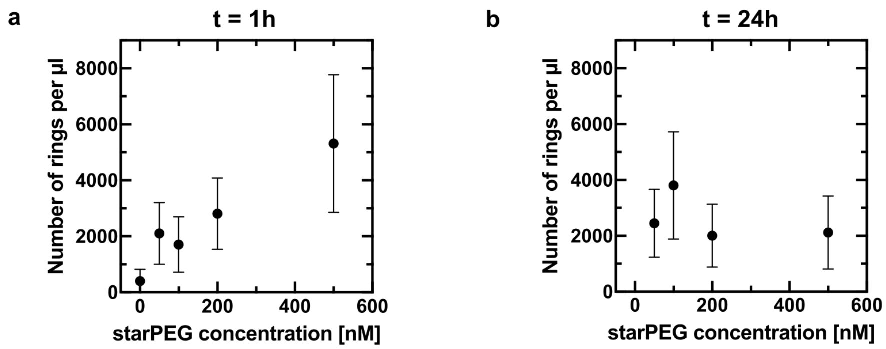

Supplementary Fig. 6: Yield of DNA rings per µL for different starPEG-$(KA7)_4$ concentrations, $30\,\mathrm{nM}$ DNA nanotubes in 1× PBS and $10\,\mathrm{mM}$ $MgCl_2$. Per condition 10 overview images $(101.41\mathrm{x}101.41\,\mu\mathrm{m}^2)$ were acquired and rings were counted $1\,\mathrm{h}$ (**a**) and $24\,\mathrm{h}$ (**b**) after sample preparation. The numbers of DNA nanotube rings per microliter are averaged over all images and their standard deviation has been calculated. Volumetric densities were calculated by measuring the chamber height $(97\,\mu\mathrm{m})$ and the image area. The displayed values are therefor shown as (Mean$\pm$SD).

Supplementary Figure 7: Microscopic structure of DNA nanotube rings

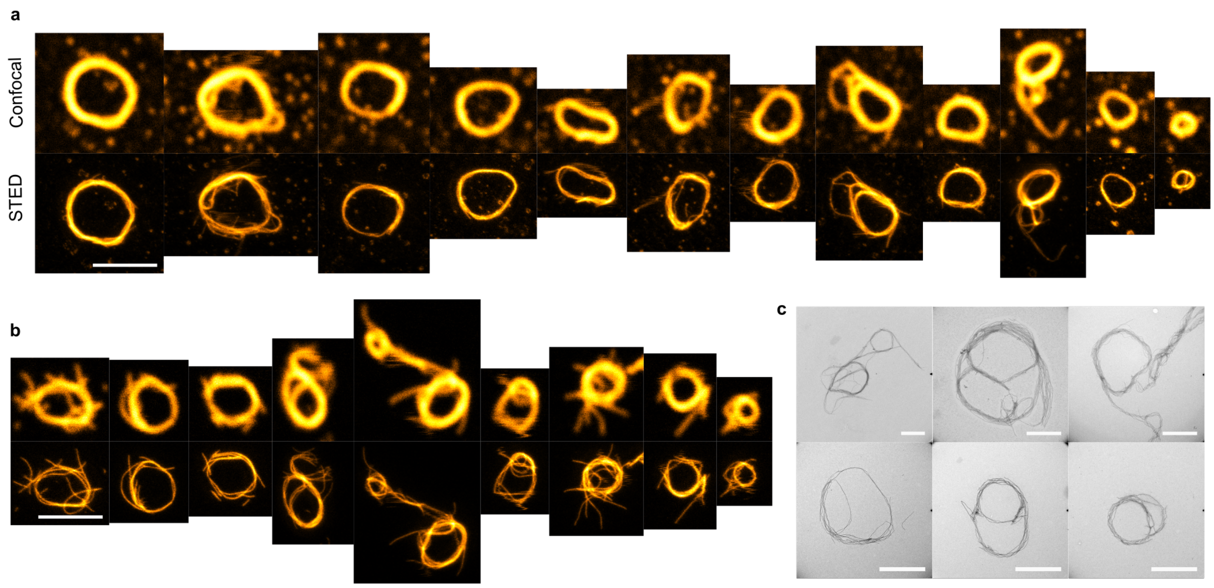

Supplementary Fig. 7: Microscopy images reveal structure of rings formed from DNA nanotube bundles. $30\,\mathrm{nM}$ tiles and $200\,\mathrm{nM}$ starPEG-(KA7)$_4$ are mixed in $1\times$ PBS and $10\,\mathrm{mM}$ $MgCl_2$. Confocal (upper row) and STED (lower row) microscopy images of DNA rings without (a) and with (b) $2.5\,\mathrm{kDa}$ dextran. Scale bars: $3\,\mu\mathrm{m}$. (c) Transmission electron micrographs of DNA rings without dextran. Scale bars: $1\,\mu\mathrm{m}$.

Supplementary Figure 8: DNA ring size dependence on $MgCl_2$ and DNA tile concentration

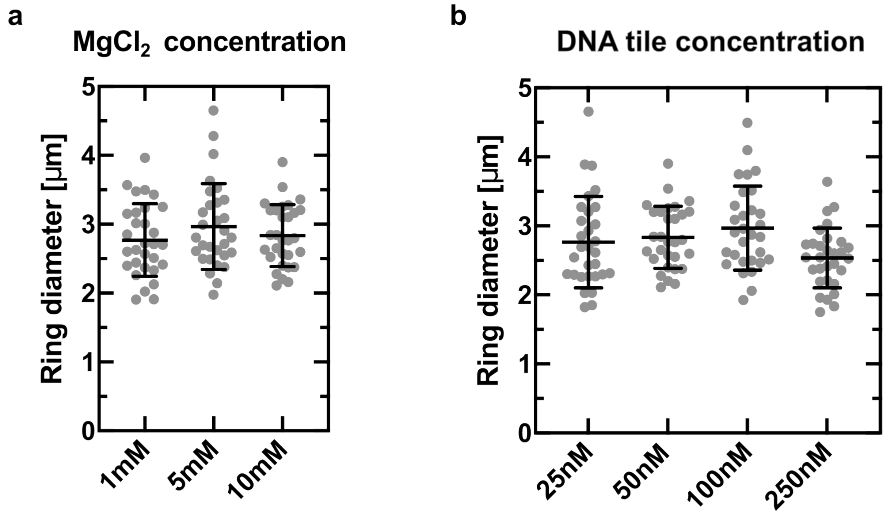

Supplementary Fig. 8: DNA ring size dependence on $MgCl_2$ and DNA tile concentration. (**a**) Ring diameter at different buffer conditions containing 1, 5 or $10\,\mathrm{mM}$ $MgCl_2$ and $50\,\mathrm{nM}$ DNA tiles while maintaining the starPEG-$(KA7)_4$:DNA tile ratio of 10:1 ($500\,\mathrm{nM}$ starPEG-$(KA7)_4$) in 1× PBS. (**b**) DNA ring diameter for different DNA nanotube concentrations maintaining a starPEG-$(KA7)_4$:DNA tile ratio of 10:1 and $10\,\mathrm{mM}$ $MgCl_2$ and 1× PBS. Per condition 30 single rings were imaged and ring diameters are plotted as Mean±SD.

Supplementary Figure 9: DNA ring diameter is time-independent

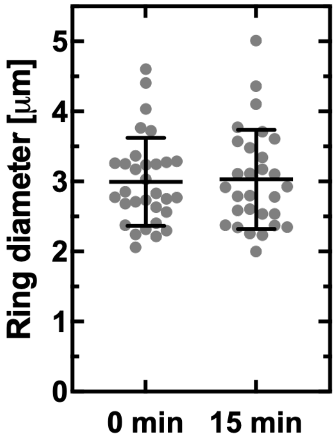

Supplementary Fig. 9: DNA ring diameter is time-independent. DNA nanotube ring diameter at room temperature after 0 and $15\,\mathrm{min}$ of incubation as used for the temperature increase experiments (Mean $\pm$ SD, $n = 29, 31$ DNA nanotube rings per condition).

Supplementary Figure 10: Analysis of the circularity of DNA rings

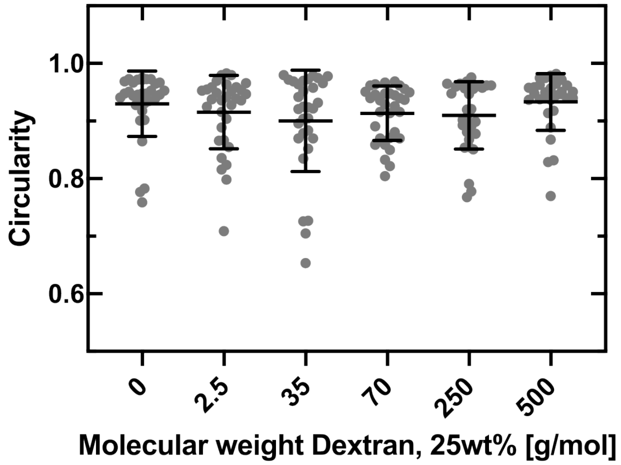

Supplementary Fig. 10: DNA ring circularity for DNA nanotubes formed from $50\,\mathrm{nM}$ DNA tiles, $500\,\mathrm{nM}$ starPEG-(KA7)$_4$ in 1x PBS and $10\,\mathrm{mM}$ $MgCl_2$ and according molecular weights $(2\ 500, 35\ 000, 70\ 000, 250\ 000,\ 500\,000\,\mathrm{g/mol})$ of dextran at $25\,\mathrm{wt\%}$ (Mean ± SD, $n = (32, 32, 31, 32, 31, 30)$ DNA nanotube rings analysed per condition).

Supplementary Figure 11: DNA ring contraction with Methylcellulose

a

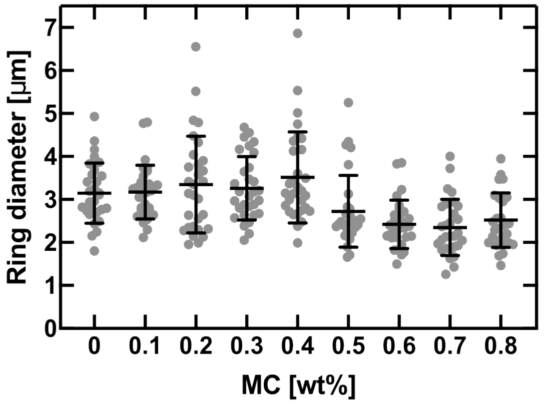

b

	0	0.1	0.2	0.3	0.4	0.5	0.6	0.7	0.8
0		Ns 0.8891	Ns 0.8660	Ns 0.6125	Ns 0.2729	** 0.0028	**** <0.0001	**** <0.0001	*** 0.0008
0.1			Ns 0.8430	Ns 0.7889	Ns 0.3601	*** 0.0007	**** <0.0001	**** <0.0001	*** 0.0002
0.2				Ns 0.8660	Ns 0.4232	* 0.0389	*** 0.0005	*** 0.0001	** 0.0017
0.3					Ns 0.4761	*** 0.0007	**** <0.0001	**** <0.0001	*** 0.0002
0.4						**** <0.0001	**** <0.0001	**** <0.0001	**** <0.0001
0.5							Ns 0.1529	Ns 0.0561	Ns 0.3637
0.6								Ns 0.5266	Ns 0.6797
0.7									Ns 0.2408
0.8									

Supplementary Fig. 11: DNA ring contraction with Methylcellulose. $25\,\mathrm{nM}$ DNA nanotubes and $250\,\mathrm{nM}$ starPEG-(KA7)$_4$ are mixed in 1× PBS and $5\,\mathrm{mM}$ $MgCl_2$ and varied concentrations of Methylcellulose. Per condition, $100\,\mu\mathrm{L}$ sample solution is freshly mixed and pipetted into a well slide (ibidi §µ-Slide 18 Well, glass bottom). (**a**) 30 rings are then imaged and analyzed using ImageJ [9] and the ring diameter is plotted (Mean±SD, $n = 30, 30, 30, 30, 30, 30, 29, 29, 29$ DNA nanotube rings per condition). (**b**) The ring diameter data from **a** is tested for significance. The corresponding p-values are obtained performing an unpaired non-parametric Mann-Whitney test.

Supplementary Tables

Supplementary Table 1: List of DNA sequences

Name	DNA sequence
SE1	CTCAGTGGACAGCCGTTCTGGAGCGTTGGACGAAACT
SE2-DIAG	GTCTGGTAGAGCACCACTGAGAGGTA
SE3	CCAGAACGGCTGTGGCTAAACAGT<br>AACCGAAGCACCAACGCT(-6-FAM/Atto633/Biotin)
SE4-EE10	CAGACAGTTTCGTGGTCATCGTACCT
SE5	CGATGACCTGCTTCGGTTACTGTTTAGCCTGCTCTAC

DNA sequences from 5’ to 3’ for single-tile DNA nanotubes, adapted from Rothemund et al. [8].

Supplementary Notes

Supplementary Note 1: Theory of bundle contraction

We present additional details of the theory of bundle contraction.

We consider a toroidal DNA nanotube bundle with radius D and a roughly circular cross section containing N DNA nanotubes resulting in a total length $L_{\text{tot}} = \pi N D$. The total length is fixed as we assume a fixed number of DNA nanotube monomers throughout contraction. This length can change by depolymerization or polymerization processes of DNA nanotubes. The bundle has a bending rigidity $\kappa_b(N)$, which is related to the bending energy κ of individual DNA nanotubes via $\kappa_b(N) = \kappa N^{\alpha}$, with $\alpha = 1$ for decoupled sliding DNA nanotubes and $\alpha = 2$ if crosslinking resists shear [4]. We assume an adhesion energy g per length for the DNA nanotubes inside the bundle. For roughly circular cross sections, there should be $N_s = a_s N^{1/2}$ out of N DNA nanotubes at the bundle surface with a geometric factor a_s of order unity (in principle, it is possible to consider a wider class of bundle cross section geometries along the lines of Schnurr et al. [10]). This results in an effective DNA nanotube length

$$L_i = L_{\text{tot}} - \pi a_s N^{1/2} D \tag{1}$$

at the interior of the DNA nanotube bundle, which is the length that is fully accessible to the attraction of strength g. Contraction of toroidal bundles can then be described by the total free energy

$$F = E_{\text{bend}} + E_{\text{ad}} + F_c \tag{2}$$

$$= 2\pi\kappa_b(N)D^{-1} - gL_i + F_c(L_i), \tag{3}$$

which is the sum of bending energy, adhesion energy at the interior and the entropic free energy F_c of the crosslinker gas at the interior of the bundle, which will arise if crosslinkers are mobile.

We neglect the entropy of DNA nanotube shape fluctuations because we focus on the bundled state; this entropy is relevant, however, for the process of bundle formation [5].

In the bundled state, the entropy of the quasi-one-dimensional gas of N_c crosslinkers distributed over the accessible bundle length L_i becomes relevant. We approximate F_c by the (entropic) free energy of a Tonks gas of N_c non-overlapping particles of crosslinker size b_c in a one-dimensional volume of length L_i with a line density of $1/b_c$ of possible binding sites,

$$F_c(L_i) = k_B T N_c \ln\left(\frac{N_c b_c}{L_i - N_c b_c}\right). \tag{4}$$

Minimizing F with respect to D gives the equilibrium diameter D of the toroidal DNA nanotube bundle,

$$0 = \frac{dE_{\text{bend}}}{dD} + \frac{dE_{\text{ad}}}{dD} + \frac{dF_c}{dD}. \tag{5}$$

We minimize at fixed L_{tot}, i.e., using $N(D) = L_{\text{tot}}/\pi D$; the effective interior bundle length to use in minimization becomes

$$L_i(D) = L_{\text{tot}} - \pi^{1/2} a_s L_{\text{tot}}^{1/2} D^{1/2}. \tag{6}$$

The bending energy will favor large D

$$\frac{dE_{\text{bend}}}{dD} = -(1+\alpha)2\pi^{1-\alpha}\kappa L_{\text{tot}}^{\alpha} D^{-\alpha-2} < 0. \tag{7}$$

Adhesion energy and crosslinker entropy favor ring contraction via increasing the overlapping interior length L_i of the bundle. The crosslinker gas increases L_i by exerting an ideal gas pressure

onto the boundaries of the overlapping region,

$$\frac{dE_{\text{ad}}}{dD} = -g\frac{dL_i}{dD} = \frac{1}{2}\pi^{1/2}a_s g L_{\text{tot}}^{1/2} D^{-1/2} > 0 \tag{8a}$$

$$\frac{dF_c}{dD} = -\frac{k_B T N_c}{L_i - N_c b_c}\frac{dL_i}{dD} \tag{8b}$$

$$= \frac{1}{2}\pi^{1/2}a_s \frac{k_B T N_c}{L_i - N_c b_c} L_{\text{tot}}^{1/2} D^{-1/2} > 0. \tag{8c}$$

Neglecting prefactors of order unity, the minimization condition (5) leads to the equilibrium diameter (Eq. (3) in the main text)

$$\left(\frac{D}{L_{\text{tot}}}\right)^{(3/2)+\alpha} \sim \frac{L_p}{L_{\text{tot}}}\frac{1}{N_c}\left(\frac{g d_c}{k_B T} + \frac{1}{1 - b_c/d_c}\right)^{-1}, \tag{9}$$

where $d_c = L_i/N_c \approx L_{\text{tot}}/N_c$ is the average distance between crosslinkers (while b_c is their minimal possible distance) and $L_p = \kappa/k_B T$ is the persistence length of individual DNA nanotubes.

Supplementary Note 2: MD simulations

Equilibration

We perform coarse-grained Molecular Dynamics (MD) simulations using LAMMPS [11] with a Langevin dynamics thermostat. DNA nanotubes are represented as bead-spring polymers with bending rigidity.

Beads interact by an attractive Lennard-Jones (LJ) interactions

$$V_{\mathrm{LJ}}(r) = \varepsilon \left((r/\sigma)^{-12} - (r/\sigma)^{-6} \right) \tag{10}$$

with a length scale σ, which represents both the range of attraction and the size of the hard core. Equilibration is impeded by the tendency of neighboring bead-spring polymers in a bundle to "lock-in" by attraction to two adjacent beads on neighboring polymers, see Supplementary Fig. 12.

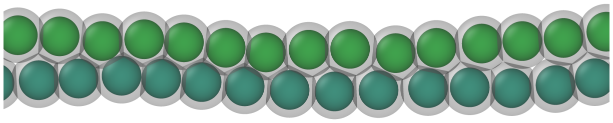

Supplementary Fig. 12: Sketch of a bundle of two mutually attractive bead-spring polymers. The transparent spheres represent the range of the LJ attraction. The beads assume a configuration where each bead has two attractive overlaps with beads from the neighboring polymer. This leads to a "locked-in" configuration, where sliding motion of beads requires activation over a barrier, where a bead has only one attractive interaction.

Bead motion along the neighboring polymer then requires activation over energy barriers, which impedes polymer sliding in simulations as the essential process for the toroidal ring to find its equilibrium diameter.

Therefore, we employ an annealing protocol to facilitate faster equilibration, where we increase the temperature to $T_2 \sim$ 2–8 T_1 for short time intervals for a simulation at temperature T_1 (measurements are always performed after switching back to temperature T_1 and after a certain waiting time at T_1). This allows the bundle to "loosen" at T_2 (because ε/k_BT_2 is reduced) such that sliding becomes possible. The value of T_2 is adapted depending on the simulated potential strength ε to prevent complete unbundling of the ring structure during the interval at T_2. For small potentials $0.2 < \varepsilon \leq 0.5\, k_BT$, an annealing temperature $T_2 = 2T_1$ is sufficient to equilibrate the rings within achievable simulation times. The annealing temperature is gradually increased with the potential up to $T_2 = 8T_1$ for $\varepsilon = 2.0\, k_BT$ because annealing at $T_2 = 2T_1$ turns out to be insufficient to "loosen" the bundle effectively for these larger values of ε. For very small $\varepsilon/k_BT_1 \leq 0.2$, we do not use simulated annealing.

We can test equilibration by starting from different initial ring diameters for the same system parameters and monitoring how long the MD simulation requires to contract rings to their common equilibrium diameter, see Supplementary Fig. 13. Equilibration in the MD simulation at a fixed temperature T_1 is fairly slow for all interaction strengths $\varepsilon/k_BT_1 \gtrsim 0.5$ well above the bundling threshold. The comparison of the different simulations in Supplementary Fig. 13 demonstrates a much more efficient equilibration with simulated annealing. For $\varepsilon/k_BT_1 = 1$ equilibration of DNA nanotube rings with parameters typical for experiments is practically arrested without annealing. For small $\varepsilon/k_BT_1 \lesssim 0.2$, however, simulated annealing should not be employed as it results in an artificial widening of the ring.

We equilibrate rings over $\sim 10^9$ MD steps and measure ring diameters in a fixed time interval between 8–10 $\times\, 10^8$ MD time steps. Fig. 14**a** shows all measured equilibration curves of ring diameters for weak attraction $\varepsilon/k_BT = 0.3$ and Supplementary Fig. 14**b** for strong attraction $\varepsilon/k_BT = 1.0$. For strong attraction and smallest bending rigidity resulting in the smallest diameters, equilibration is most difficult because of the lock-in of neighboring polymers. The

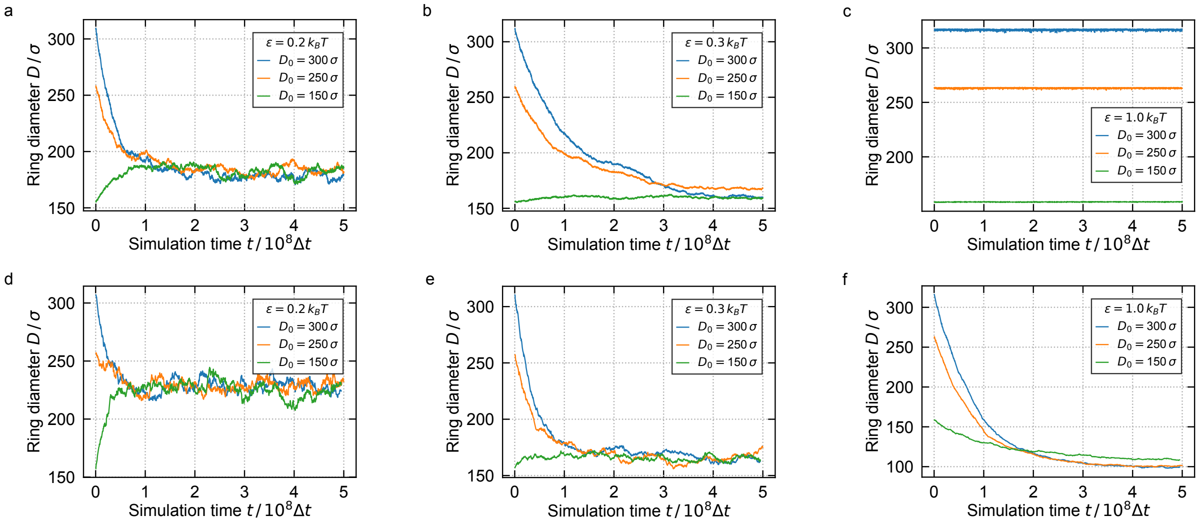

Supplementary Fig. 13: Comparison of equilibration dynamics by monitoring ring contraction from three different initial ring diameter to the final identical equilibrium diameter. All plots show ring diameter as a function of simulation time. Top row: Standard MD simulations for a fixed temperature k_BT_1 for different interactions $\varepsilon/k_BT_1 = 0.2,\ 0.3,\ 1.0$ (from left to right). Equilibration is only reached for small $\varepsilon/k_BT_1 = 0.2,\ 0.3$. Bottom row: MD simulations employing simulated annealing, where we increase the temperature to $T_2 \sim 2T_1$ (for $\varepsilon/k_BT_1 = 0.2,\ 0.3$) or $T_2 \sim 4T_1$ (for $\varepsilon/k_BT_1 = 1.0$) for short time intervals. This allows us to achieve equilibration also for larger $\varepsilon/k_BT_1 = 1.0$. For small $\varepsilon/k_BT_1 = 0.2$, however, simulated annealing results in an artificial widening of the ring.

lowest curves in the Supplementary Fig. 14**b** show the resulting bundle contraction during equilibration in this regime.

Extended simulation runs up to 2×10^9 MD simulation steps actually show that the lowest three simulations are not fully equilibrated and the ring diameter is still decreasing at the end of the simulations. This explains the relatively large deviation of the first three light yellow crosses in Fig. 5**c** in the main text from the scaling law (the time interval for ring diameter measurements is shifted to $18-20 \times 10^8\,\Delta t$ for these simulations). The annealing procedure is less effective for strong attraction and small bending rigidity because small bending rigidities reduce the bending moments expanding the ring after annealing has effectively weakened the attraction by thermal shape fluctuations. This considerably slows down "loosening" of the bundle during annealing.

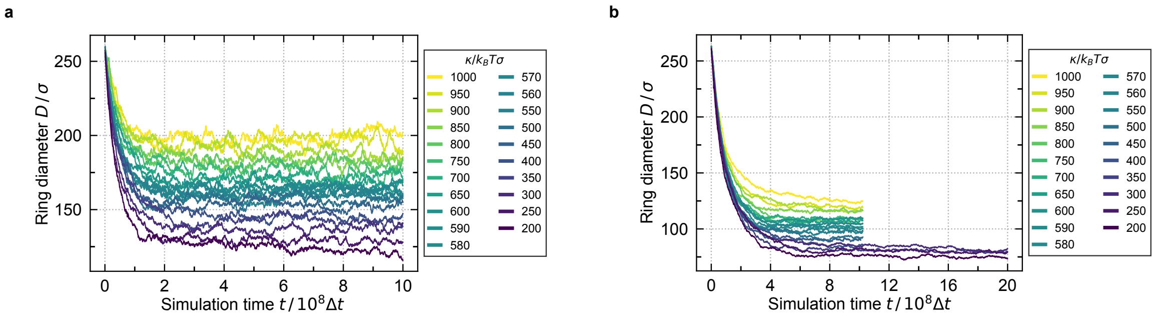

Supplementary Fig. 14: Bundle contraction during equilibration (ring diameter as a function of MD simulation time) for different single DNA nanotube rigidities κ for **a**, $\varepsilon/k_BT = 0.3$ (showing data for more κ-values as compared to Fig. 5**a** in the main text) and **b**, $\varepsilon/k_BT = 1.0$.

Time scales of MD simulation

The natural time scale of the LAMMPS MD simulation is the Lennard-Jones times scale $\tau_{\mathrm{LJ}} = \sqrt{m\sigma^2/\varepsilon}$. This is the time scale over which the inertial force $\vec{F}_i = m\ddot{\vec{r}}_i$ of a bead becomes comparable to the typical Lennard-Jones force between beads. The mass m of a DNA nanotube piece of length $\sigma \approx 12\mathrm{nm}$ is $m \approx 240\mathrm{kDa}$ using a mass density $\lambda \approx 20\mathrm{kDa/nm}$ [8]. For $\varepsilon = 1k_BT$, this gives a Lennard-Jones time scale $\tau_{\mathrm{LJ}} \approx 3.8\,\mathrm{ns}$. The actual LAMMPS MD simulation time step Δt is chosen much smaller than τ_{LJ} to assure accurate results, $\Delta t = 5 \times 10^{-3}\,\tau_{\mathrm{LJ}} \approx 0.02\,\mathrm{ns}$.

Using the Langevin thermostat we introduce an additional friction force $\vec{F}_f = -\gamma m\dot{\vec{r}}$ with a friction coefficient $\gamma = 3\pi\sigma\eta/m$ for each bead and a corresponding thermal noise satisfying the Einstein relation. Here, η is the viscosity of the surrounding medium. The friction force sets a frictional time scale $\tau_\gamma = \gamma^{-1} \propto \eta^{-1}$, over which the inertial force $\vec{F}_i$ of a bead becomes comparable to the friction force such that for times $t > \tau_\gamma$ the motion becomes overdamped.

The equilibration process during which rings contract to their final equilibrium diameter takes up to $\sim 10^9$ MD steps for the smallest ring diameters. This corresponds to a simulation time $t = 10^9\Delta t = 20\,\mathrm{ms}$. To achieve such a fast equilibration we employ, however, viscosities η

much lower than the realistic viscosity of water, $\eta \approx 10^{-5}\eta_{\text{Water}}$ or $\tau_\gamma = 100\tau_{\text{LJ}} \approx 10^5\tau_{\gamma,\text{Water}}$.

We argue that this will correspond to equilibration times

$$t_{\text{Water}} \sim \min\left(\frac{\tau_{\text{LJ}}}{\tau_\gamma(\eta)}, 1\right)\frac{\eta_{\text{Water}}}{\eta}t \approx 10^3 t \approx 20\,\text{s} \tag{11}$$

if we could simulate with the high viscosity of water. For high viscosities such that $\tau_\gamma(\eta) < \tau_{\text{LJ}}$, the simulation is overdamped already on the Lennard-Jones time scale such that inertia can be neglected, and we essentially have a Brownian dynamics. For the viscosity of water, we have $\tau_\gamma(\eta_{\text{Water}}) = 10^{-3}\tau_{\text{LJ}}$ and are deep in the overdamped limit (as is typical for all biological systems on the micron scale). Within the overdamped Brownian regime, the equilibration time t scales linearly with viscosity $t \sim \eta$ such that $t_{\text{Water}} \sim (\eta_{\text{Water}}/\eta)t$ because all motion is slowed down proportional to friction. This can be shown strictly by non-dimensionalizing time in the Brownian equation of motion in units of τ_γ, which completely removes viscosity from the equation of motion. For lower viscosities such that $\tau_\gamma(\eta) > \tau_{\text{LJ}}$, on the other hand, the simulation is underdamped and the equilibration time t becomes essentially independent of viscosity. Combining the findings for overdamped and underdamped regimes leads to the above overall result (11) for the equilibration time at different viscosities.

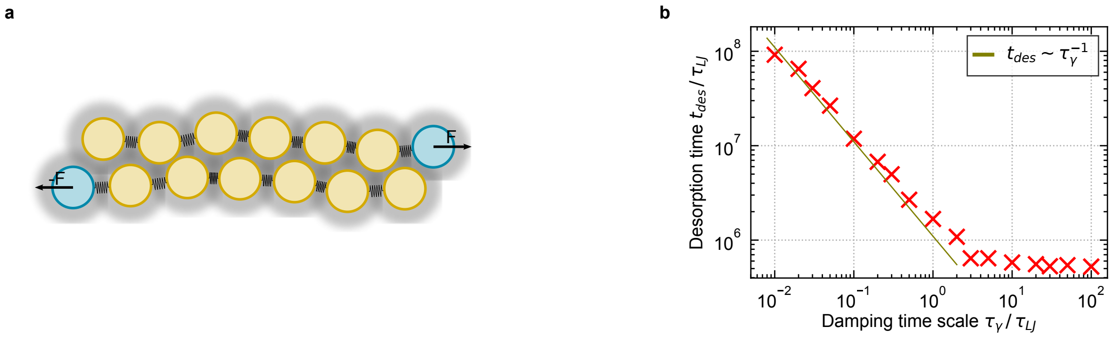

Supplementary Fig. 15: **a**, MD simulation of two bundled polymers sliding apart by pulling with a force F. Polymers have 580 beads, the pulling force is $F = 1k_BT/\sigma$, and the interaction energy is $\varepsilon/k_BT = 0.5$. **b**, Desorption time t_{des} in units of τ_{LJ} as a function of the damping time scale $\tau_\gamma(\eta)/\tau_{\text{LJ}} \propto 1/\eta$.

To further support this argument we performed MD simulations of a faster process than ring contraction, namely the pulled sliding of only two bundled polymers with a fixed external pulling force F, see Fig. 15**a**, where we consider the desorption time t_{des} to separate both polymers. This process is faster, such we can explore a wider range of viscosities in MD simulations. According to our arguments leading to Eq. (11), the total desorption time t_{des} should scale linearly with viscosity in the overdamped regime $\tau_\gamma(\eta) < \tau_{\text{LJ}}$ and become essentially independent of viscosity in the underdamped regime $\tau_\gamma(\eta) > \tau_{\text{LJ}}$,

$$t_{\text{des}}/\tau_{\text{LJ}} \propto \max\left(1, \frac{\tau_{\text{LJ}}}{\tau_\gamma(\eta)}\right) . \tag{12}$$

Extrapolating desorption times from a lower viscosity η to the high viscosity of water with $\tau_\gamma(\eta_{\text{Water}}) < \tau_{\text{LJ}}$ gives again

$$\frac{t_{\text{des}}(\eta_{\text{Water}})}{t_{\text{des}}(\eta)} = \frac{\tau_{\text{LJ}}/\tau_\gamma(\eta_{\text{Water}})}{\max\left(1, \tau_{\text{LJ}}/\tau_\gamma(\eta)\right)} = \min\left(\frac{\tau_{\text{LJ}}}{\tau_\gamma(\eta)}, 1\right)\frac{\eta_{\text{Water}}}{\eta} \tag{13}$$

in agreement with Eq. (11).

In Supplementary Fig. 15**b**, we measure the desorption time as a function of the damping time scale $\tau_\gamma(\eta)/\tau_{\text{LJ}}$ and fully confirm Eq. (12): we find a decrease $\propto (\tau_\gamma(\eta)/\tau_{\text{LJ}})^{-1}$ corresponding to a linear increase with viscosity in the overdamped regime $\tau_\gamma(\eta) < \tau_{\text{LJ}}$, and an essentially η-independent desorption rate in the underdamped regime $\tau_\gamma(\eta) > \tau_{\text{LJ}}$.

Ring formation

In a different set of simulations we aim to follow the process of ring formation starting from a solution of polymers. To observe the process of ring formation on computationally accessible time scales, we employ a reduced bending stiffness because the bending energy enters the time scale for ring closure in an Arrhenius-type exponential dependence. We use $\kappa = 200 k_B T\sigma$ as compared to the realistic stiffness of $\kappa = 600 k_B T\sigma$ for DNA nanotubes. Fig. 4**c** in the main text

shows a sequence of snapshots of a ring formation simulation. We find that ring formation in simulations is optimal for an initial small bundle containing few DNA nanotubes such that its total contour length is $\sim 3L_p$. For all MD simulations of ring contraction (Fig. 5 in the main text), we enhance initial ring formation by initially positioning DNA nanotubes in a ring-like structure ´and employ realistic bending stiffnesses.

Measuring ring diameters

In order to calculate the ring diameters of DNA nanotube bundles in simulations we compute the 3x3 gyration tensor

$$S_{mn} = N_{\text{tot}}^{-1} \sum_{i=1}^{N_{\text{tot}}} r_m^{(i)} r_n^{(i)} \tag{14}$$

by averaging over all bead positions $\vec{r}^{(i)}$. The tensor is symmetric and has three eigenvalues X^2, Y^2 and Z^2. For a homogeneous torus with major axis b and minor axis a, we can calculate the eigenvalues of the gyration tensor as

$$X^2 = \frac{1}{4}a^2,\ Y^2 = Z^2 = \frac{1}{2}\left(b^2 + \frac{3}{4}a^2\right). \tag{15}$$

Therefore, we calculate the eigenvalues X^2, Y^2, and Z^2 of the measured tensor S in a toroidal ring configuration numerically and identify

$$D^2 = 4b^2 = 4Y^2 + 4Z^2 - 12X^2 \tag{16}$$

as ring diameter D.

Supplementary Note 3: Discussion of ring contraction-driving parameters for physical system

Temperature dependence of parameters

According to the theoretical result (9) the ring diameter is controlled by several parameters that can be controlled experimentally and in simulations and that are possibly temperature dependent:

- The bending rigidity κ appears via the corresponding persistence length $L_p = \kappa/k_BT$ (which measures κ in units of k_BT). The bending rigidity κ is assumed to be temperature independent (both in experiments and simulations) such that the persistence length $L_p \propto 1/T$ by definition.

- The (free) adhesion energy g per length for the DNA nanotubes inside the bundle appears as g/k_BT, which is an inverse length measuring g in units of k_BT. The free energy of adhesion g is temperature-dependent in general.

  Close to the bundling threshold it will contain entropic contributions from shape fluctuations of the DNA nanotubes [5]. This complication is absent sufficiently deep in the bundled phase; this is the limit we focus on in experiments and simulations. Additional temperature dependencies can arise depending on the nature of the interaction.

  Crosslinker-mediated adhesion can be assumed to lead to a largely temperature independent g. The only temperature dependence stems from the unbinding and rebinding equilibrium of crosslinking molecules [5]. For a fixed number N_c of firmly bound crosslinkers, this temperature-dependence is absent. Then $g/k_BT \propto 1/T$. This is the relevant situation for crosslinked DNA nanotube bundles.

  Depletion attraction by additional crowding agents is an entropic interaction with a linear temperature-dependence $g \propto k_BT$ such that g/k_BT becomes temperature independent.

This is relevant for the additional depletion attraction triggering ring contraction.

In simulations, we employ a temperature independent fixed g, which is given by a temperature independent parameter ε via $g \approx \varepsilon/\sigma$.

- The total contour length L_{tot} is assumed to be temperature independent, since we neglect the possibility of melting. Therefore, also the average crosslinker distance $d_c = L_i/N_c \approx L_{\text{tot}}/N_c$ is temperature independent (for fixed number N_c of crosslinkers).

- Geometric parameters such as the crosslinker size b_c are temperature independent.

Conclusion for the physical system from theoretical predictions

The theoretical result (Eq. 9) predicts several experimentally testable scenarios under which ring contraction could occur given the temperature dependence of the parameters:

(i) Rings contract for decreasing persistence length or bending rigidity, $D \propto L_p^{1/(3/2+\alpha)}$ with $L_p = \kappa/k_B T$.

In experiments, a decrease in persistence length (i) could be achieved by increasing the temperature, as temperature is known to decrease the persistence length of semiflexible polymers and in particular DNA [2]. As an experimental control, the persistence length of the DNA nanotube bundle can be increased by depletion when first adding a molecular crowder, like dextran, and second adding the crosslinker starPEG-(KA7)$_4$ in order to form larger rings. In the simulation experiments, the bending stiffness $\kappa/k_B T\sigma$ can be altered directly.

(ii) Rings contract if the total DNA nanotube length is decreased, for example, by depolymerization, $D \propto L_{\text{tot}}^{(1/2+\alpha)/(3/2+\alpha)}$.

In experiments, we have to consider the melting of DNA from its double stranded into its single stranded state. The critical melting temperature for DNA nanotubes can be calculated as a function of the enthalpy of disassembly, the entropy, the number of sticky end bonds and base pairs per bond and the concentration of free tiles [3, 12]. Decreasing free tile concentrations lower the corresponding melting temperature of a DNA nanotube in solution. Assuming $50\,\mathrm{nM}$ final tile concentration (of assembled DNA nanotubes, they are pre-formed at $5\,\mu\mathrm{M}$ and then diluted 1:100), one could gently estimate the concentration of free tiles to be below $5\,\mathrm{nM}$. This would result in a melting temperature of $37.2\,^{\circ}\mathrm{C}$ (compare to $50\,\mathrm{nM}$ free tiles resulting in $42.0\,^{\circ}\mathrm{C}$).

In addition, experiments have shown that in absence of free tiles, the depolymerization rate measures around 0.3 layers per second at $40\,^{\circ}\mathrm{C}$ [3]. Moreover, the gradual disassembly of DNA nanotubes confined in water-in-oil droplets has been shown for temperatures above room temperature [13].

At the interior of the bundle, the concentration of free tiles is higher (due to recent depolymerization and depletion effects), leading to an effective increase of the melting temperature. A moderate increase in temperature will thus likely lead to depolymerization of the loose ends of the DNA nanotubes that were seen experimentally in STED (Fig. 3**b**, Supplementary Fig. 7) and electron microscopy (Fig. 4**e**) as well as in simulation experiments (Fig. 4**c**, Fig. 4**d**, Supplementary Movies 2,3) . DNA nanotube depolymerization can thus enhance ring contraction further. However, depolymerization is not included in the simulations. For simplicity L_{tot} is kept constant.

(iii) If crosslinker entropy can be neglected, the ring diameter will contract with increasing g according to $D \propto (\kappa/g)^{1/(3/2+\alpha)}$. This means that if g is an entropic depletion attraction with $g \propto T$ ring contraction occurs for increasing temperature or decreasing persistence

length of individual DNA nanotubes, $D \propto T^{-1/(3/2+\alpha)} \propto L_p^{1/(3/2+\alpha)}$ (iii.a). If g is a crosslinker-mediated attraction, D is independent of temperature although the persistence length will decrease (iii.b).

For a purely crosslinked system (only starPEG-(KA7)$_4$, no dextran) the DNA nanotube colocalization assay indicates that nanotube-nanotube interaction relies on specific crosslinking (Fig. 2**a,b**). Moreover, increasing starPEG-(KA7)$_4$ concentration alone did not contract the ring size (Fig. 3**c**), indicating that assumption (iii.b) is not valid on its own. Experimentally, g can be increased by an additional depletion force (iii.a). The addition of dextran could shed light on the contraction for increasing attraction strength g and, furthermore, on the temperature dependence of the contractile system.

(iv) For dominant crosslinker entropy, rings will contract with increasing temperature or decreasing persistence length, $D \propto T^{-1/(3/2+\alpha)} \propto L_p^{1/(3/2+\alpha)}$. The crosslinker entropy is dominant for $gd_c < k_BT/(1 - b_c/d_c)$, where gd_c is the average adhesion energy per crosslinker; in particular, it becomes dominant for a dense crosslinker gas with $b_c/d_c \leq 1$ if the average adhesion per crosslinker is of the order of several k_BT. An additional entropic depletion attraction with $g \propto T$ will further contract rings under these conditions (iv.a).

In our experimental system (iv) would indicate that at low crosslinker concentrations temperature increase would not cause crosslinker entropy to constrict the ring size. However, since this setting cannot be completely isolated experimentally from crosstalk effects, we can only make sure that we are in the parameter range of a dense crosslinker gas to obtain efficient constriction. Then (iv.a) predicts ring contraction for increasing temperature *and* further ring contraction upon increasing the depletion attraction, for example, by increasing the depletant concentration or the molecular size of depletants.

In summary, the key parameters for ring constriction are L_p and g. These can be controlled in

simulations as well as experiments with DNA nanotube rings, making the theoretical predictions testable.

Supplementary Note 4: Molecular weight dependence of dextran-induced depletion attraction

Addition of molecular crowders gives rise to an additional depletion attraction between DNA nanotubes. For two long *hard* rods of diameter d, the depletion interaction energy per rod from a molecular crowder with a depletion length δ around the rod is [6]

$$g_{\text{dep}} = k_B T \frac{c}{M} A_{\text{ov}}(\delta, d), \tag{17}$$

where c is the mass concentration of a crowder of molecular mass M and A_{ov} is the overlap area of the circular cross sections of two rods of diameter $d + 2\delta$ and center distance d,

$$\begin{aligned} A_{\text{ov}} &= \frac{1}{2}(d+2\delta)^2 \left(\varphi - \cos\varphi \sin\varphi\right) \quad \text{with} \\ \cos\varphi &= \frac{d}{d+2\delta}. \end{aligned} \tag{18}$$

The depletion length δ is the size of the depletion layer and will be related to the size of the molecular crowders below. In the limits of small ($\delta \ll d$) or large ($\delta \gg d$) depletion length we obtain $\varphi \approx 2\sqrt{\delta/d} \ll 1$ and $\varphi \approx \pi/2$, respectively, leading to

$$A_{\text{ov}} \approx \begin{cases} \frac{8}{3} d^{1/2} \delta^{3/2} & (\delta \ll d), \\ \pi \delta^2 & (\delta \gg d). \end{cases} \tag{19}$$

To a good approximation, dextran can be considered as an ideal polymer with a gyration radius $R_g \propto M^{1/2}$: In Fragasso et al. [1], $R_g = R_0 M^{0.48}$ (with $R_0 = 0.072\,\text{nm}$ and M in Da), has been measured.

For polymers, the depletion length δ is defined as the depletion layer thickness via $\delta = \int_0^\infty dx(1 - f(x))$, where x is a coordinate measuring the distance from a flat surface and $f(x) \in [0, 1]$ the relative polymer segment distribution [6]. For ideal polymers, the depletion

length is essentially given by the radius of gyration R_g [6, 7]; we use $\delta \approx 1.4 R_g$ [7] resulting in

$$\delta = 1.4 R_g = 1.4 R_0 M^{1/2} \tag{20}$$

Using this in (19) and (17), we find a depletion attraction

$$g_{\text{dep}} \sim \begin{cases} k_B T c d^{1/2} R_0^{3/2} M^{-1/2} & (R_g \ll d), \\ k_B T c R_0^2 & (R_g \gg d), \end{cases} \tag{21}$$

between hard rods, i.e., the depletion attraction *decreases* with molecular weight M before it saturates for large M in the regime $R_g \gg d$ (see Fig. 16, $p = 0$).

For crosslinked DNA nanotubes, on the other hand, we see stronger ring contraction for larger molecular weights (Fig. 5 main text), which points at an *increasing* depletion attraction with molecular weights. The reason for this behavior are the crosslinkers, which act as a penetrable layer of thickness p around the DNA nanotubes, and play a similar role as the soft glycocalix for the aggregation of red blood cells by dextran [7]. For a *soft* rod of total diameter d with a penetrable layer of thickness p, the depletion attraction is modified to

$$g_{\text{dep}} = k_B T \frac{c}{M} A_{\text{ov}}(\delta - p, d). \tag{22}$$

Switching from a energy per length g_{dep} to an energy per bead $\varepsilon_{\text{dep}} \approx g_{\text{dep}}\sigma$ used in our MD simulations (with $\sigma = d$), we obtain

$$\begin{aligned} \frac{\varepsilon_{\text{dep}}}{k_B T} &= d \frac{c}{M} A_{\text{ov}}(\delta - p, d) \\ &\approx \begin{cases} d \frac{c}{M} \frac{8}{3} d^{1/2} (1.4 R_0 M^{1/2} - p)^{3/2} & (R_g \ll d), \\ d \frac{c}{M} \pi (1.4 R_0 M^{1/2} - p)^2 & (R_g \gg d). \end{cases} \end{aligned} \tag{23}$$

For $p > 0$ the depletion attraction only sets in for $\delta > p$ above a threshold molecular weight M. We write the total effective diameter d of a crosslinker-decorated DNA nanotube as $d = d_0 + 2p$,

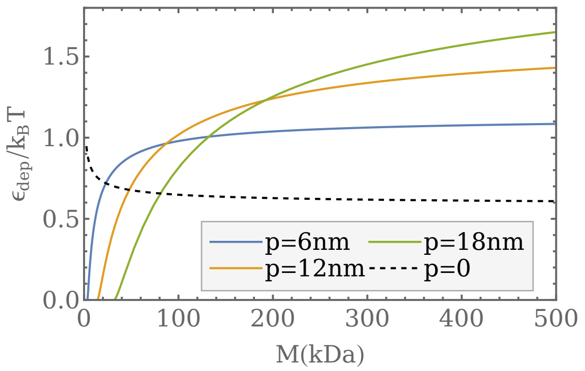

Supplementary Fig. 16: Additional depletion attraction $\varepsilon_{\text{dep}}/k_BT$ as a function of dextran molecular weight M (in kDa) for different thicknesses p of penetrable layer around a DNA nanotube of diameter $d_0 = 12\,\text{nm}$. The dextran concentration is 25 wt% $= 1.5 \times 10^{-3}\,\text{kDa}/\text{nm}^3$.

where the crosslinkers act as a penetrable layer of thickness p and $d_0 = 12\,\text{nm}$ is the bare diameter of the DNA nanotubes, which are regarded as inpenetrable. The depletion attraction (23) typically *increases* with molecular weight M (for $p \gtrsim 2\,\text{nm}$) before it saturates for large M in the regime $R_g \gg d$ (see Fig. 16, $p = 6, 12, 18\,\text{nm}$) to $\varepsilon_{\text{dep}}/k_BT \approx 2cdR_0^2$.

Using the fit $D/\sigma = a(\varepsilon/k_BT)^{-2/5}$ with a fit parameter $a \approx 100$ for the dependence of the ring diameter D on attraction from our MD simulation results (for fixed $\kappa/k_BT\sigma = 600$, blue circles in Fig. 4**e** in the main text) together with equation (23) and $\sigma = d_0$, we can obtain predictions for the ring diameter as a function of depletant molecular weight,

$$D = 100d_0\left((\varepsilon_{\text{cross}} + \varepsilon_{\text{dep}}(M))/k_BT\right)^{-2/5}. \tag{24}$$

With a crosslinker contribution $\varepsilon_{\text{cross}} = 0.3k_BT$, $d = d_0 + 2p$ as effective diameter of a crosslinker-decorated DNA nanotube, and $d_0 = 12\,\text{nm}$, we obtain the results in Fig. 17 with a steep decrease of the ring radius for $M \lesssim 50\,\text{kDa}$ and a saturation for $M \gtrsim 50\,\text{kDa}$.

Fig. 5d in the main text shows a weighted least-square fit of the experimental data with $d = d_0 + 2p$ and three fit parameters: the bare DNA nanotube diameter d_0, the penetration depth

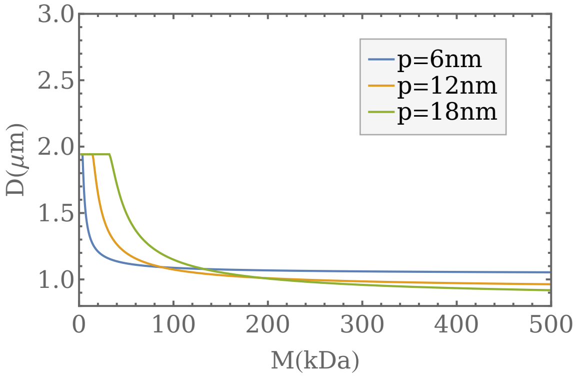

Supplementary Fig. 17: Ring diameter as a function of dextran molecular weight M (in kDa) for different thicknesses p of penetrable layer around a DNA nanotube of diameter $12\,\mathrm{nm}$ according to MD simulations with $\varepsilon = 0.3k_BT + \varepsilon_{\text{dep}}(M)$. The dextran concentration is 25 wt% $= 1.5 \times 10^{-3}\,\mathrm{kDa/nm}^3$.

p indicating the size of the penetrable crosslinker layer, and the crosslinker contribution $\varepsilon_{\text{cross}}$ to the attraction. The resulting fit values are $d_0 = 18.58 \pm 2.38$nm, $p = 11.03 \pm 3.28$nm, and $\varepsilon_{\text{cross}}/k_BT = 0.31 \pm 0.14$.

Supplementary References

[1] Alessio Fragasso et al. "Reconstitution of Ultrawide DNA Origami Pores in Liposomes for Transmembrane Transport of Macromolecules". In: *ACS Nano* 15.8 (2021), pp. 12768–12779. ISSN: 1936086X. DOI: 10.1021/acsnano.1c01669.

[2] Stephanie Geggier, Alexander Kotlyar, and Alexander Vologodskii. "Temperature dependence of DNA persistence length". In: *Nucleic Acids Research* 39 (4 Mar. 2011), pp. 1419–1426. ISSN: 03051048. DOI: 10.1093/nar/gkq932.

[3] Rizal F. Hariadi, Bernard Yurke, and Erik Winfree. "Thermodynamics and kinetics of DNA nanotube polymerization from single-filament measurements". In: *Chemical Science* 6 (4 Apr. 2015), pp. 2252–2267. ISSN: 20416539. DOI: 10.1039/c3sc53331j.

[4] Claus Heussinger, Mark Bathe, and Erwin Frey. "Statistical Mechanics of Semiflexible Bundles of Wormlike Polymer Chains". In: *Phys. Rev. Lett.* 99.4 (July 2007), p. 048101. ISSN: 0031-9007. DOI: 10.1103/PhysRevLett.99.048101.

[5] Jan Kierfeld, Torsten Kühne, and Reinhard Lipowsky. "Discontinuous unbinding transitions of filament bundles". In: *Phys. Rev. Lett.* 95.3 (July 2005), p. 038102. ISSN: 00319007. DOI: 10.1103/PhysRevLett.95.038102.

[6] Henk N.W. Lekkerkerker and Remco. Tuinier. *Colloids and the Depletion Interaction*. Vol. 833. Lecture Notes in Physics. Dordrecht: Springer Netherlands, 2011. ISBN: 978-94-007-1222-5. DOI: 10.1007/978-94-007-1223-2.

[7] Björn Neu, Rosalinda Wenby, and Herbert J. Meiselman. "Effects of Dextran Molecular Weight on Red Blood Cell Aggregation". In: *Biophys. J.* 95.6 (Sept. 2008), pp. 3059–3065. ISSN: 00063495. DOI: 10.1529/biophysj.108.130328.

[8] Paul W. K. Rothemund et al. “Design and Characterization of Programmable DNA Nanotubes”. In: *Journal of the American Chemical Society* 126.50 (Nov. 2004), pp. 16344–16352. DOI: 10.1021/ja044319l.

[9] Johannes Schindelin et al. “Fiji: an open-source platform for biological-image analysis”. In: *Nature methods* 9.7 (2012), pp. 676–682.

[10] B. Schnurr, F. Gittes, and F. C. MacKintosh. “Metastable intermediates in the condensation of semiflexible polymers”. In: *Phys. Rev. E* 65.6 (June 2002), p. 061904. ISSN: 1063-651X. DOI: 10.1103/PhysRevE.65.061904.

[11] A. P. Thompson et al. “LAMMPS - a flexible simulation tool for particle-based materials modeling at the atomic, meso, and continuum scales”. In: *Comp. Phys. Comm.* 271 (2022), p. 108171. DOI: 10.1016/j.cpc.2021.108171.

[12] Erik Winfree. *Simulations of Computing by Self-Assembly*. California Institute of Technology, 1998. DOI: 10.7907/Z9TB14X7.

[13] Pengfei Zhan et al. “Functional DNA-based cytoskeletons for synthetic cells”. In: *Nature Chemistry* 14 (June 2022), pp. 958–963. ISSN: 1755-4330. DOI: 10.1038/s41557-022-00945-w.
