# Supplementary material for: Triggered contraction of self-assembled micron-scale DNA nanotube rings
Source: Nat Commun. 2024 Mar 14;15:2307. doi: 10.1038/s41467-024-46339-z (PMC10940629; doi:10.1038/s41467-024-46339-z)
Supplement: Supplementary file 3 — Description of Additional Supplementary Files [file 41467_2024_46339_MOESM3_ESM.pdf]

Description of Additional Supplementary Files

Supplementary Movies

Supplementary Movie 1: Freely moving DNA nanotube ring
Confocal time stack of a freely moving DNA nanotube ring formed from 50 nM DNA tiles, 500 nM starPEG-(KA7)4-TAMRA in 1x PBS and 10 mM $MgCl_2$. Scale bar: 10 µm.

Supplementary Movie 2: Simulated DNA nanotube ring in three-dimensional box (all nanotubes colored)
Simulated equilibration and contraction of a DNA nanotube ring. All DNA nanotubes are colored.The movie consists of snapshots taken from the simulation shown in the main text (Fig. 5b) for the simulation parameters interaction strength $\varepsilon/k_BT = 0.2$ and bending rigidity $\kappa/k_BT\ \sigma = 600$ and includes simulation times $t/\Delta t \leq 108$. A box of side length 300 σ is depicted to provide a reference length scale, the actual simulation volume is considerably larger (side length 800 σ). To suppress diffusive translational and rotational motion of the ring, all particle positions are transformed to an eigenbasis of the ring with the center-of-mass as origin. The eigenbasis is constructed from the eigenvector corresponding to the smallest eigenvalue of the gyration tensor (out of ring-plane) and the projection of the relative position of one (in principle arbitrary) particle on the ring-plane defined by the remaining two eigenvectors of the gyration tensor. A third basis vector is then readily constructed. A simple expansion of the coordinates with respect to the eigenvectors of the gyration tensor is not suitable to construct a temporally persistent eigensystem because the degeneracy of the eigenvalues associated with in-plane eigenvectors allows these eigenvectors to rotate.

Supplementary Movie 3: Simulated DNA nanotube ring in three-dimensional box (single nanotubes colored)
Simulated equilibration and contraction of a DNA nanotube ring. For better visibility of the sliding process only selected DNA nanotubes are colored. The movie visualizes the same simulation as Supp. Movie 2 (interaction strength $\varepsilon/k_BT = 0.2$, bending rigidity $\kappa/k_BT\ \sigma = 600$ from main text Fig. 5b) and an identical coordinate transformation is performed. Box: 300 σ × 300 σ.
