# Supplementary material for: Triggered contraction of self-assembled micron-scale DNA nanotube rings
Source: Nat Commun. 2024 Mar 14;15:2307. doi: 10.1038/s41467-024-46339-z (PMC10940629; doi:10.1038/s41467-024-46339-z)
Supplement: Supplementary file 7 — Reporting Summary [file 41467_2024_46339_MOESM7_ESM.pdf]

nature portfolio

Corresponding author(s): Kerstin Göpfrich, Jan Kierfeld, Stefan Diez

Last updated by author(s): Mar 4, 2024

Reporting Summary

Nature Portfolio wishes to improve the reproducibility of the work that we publish. This form provides structure for consistency and transparency in reporting. For further information on Nature Portfolio policies, see our Editorial Policies and the Editorial Policy Checklist.

Statistics

For all statistical analyses, confirm that the following items are present in the figure legend, table legend, main text, or Methods section.

n/a	Confirmed	
☐	☒	The exact sample size (n) for each experimental group/condition, given as a discrete number and unit of measurement
☐	☒	A statement on whether measurements were taken from distinct samples or whether the same sample was measured repeatedly
☐	☒	The statistical test(s) used AND whether they are one- or two-sided<br>*Only common tests should be described solely by name; describe more complex techniques in the Methods section.*
☒	☐	A description of all covariates tested
☒	☐	A description of any assumptions or corrections, such as tests of normality and adjustment for multiple comparisons
☐	☒	A full description of the statistical parameters including central tendency (e.g. means) or other basic estimates (e.g. regression coefficient) AND variation (e.g. standard deviation) or associated estimates of uncertainty (e.g. confidence intervals)
☐	☒	For null hypothesis testing, the test statistic (e.g. F, t, r) with confidence intervals, effect sizes, degrees of freedom and P value noted<br>*Give P values as exact values whenever suitable.*
☒	☐	For Bayesian analysis, information on the choice of priors and Markov chain Monte Carlo settings
☒	☐	For hierarchical and complex designs, identification of the appropriate level for tests and full reporting of outcomes
☒	☐	Estimates of effect sizes (e.g. Cohen's d, Pearson's r), indicating how they were calculated

Our web collection on statistics for biologists contains articles on many of the points above.

Software and code

Policy information about availability of computer code

Data collection	Zen software (Version 3.1), cSeries Capture Software (Azure Biosystems C600), Electron microscopy (FEI TEM Imaging and Analysis TIA (Version 4.7 SP3))<br>The code that supports the simulation related data of this study is available in https://doi.org/10.5281/zenodo.10728688.
Data analysis	Microsoft Excel (Version 16.60), ImageJ (Version 2.3.0/1.5q), Inkscape (Version 1.1.2), Prism Graphpad (Version 9.1.2, 9.2.0 (332), 9.4.0), Python (Version 3.9), LAMMPS (Version 2020 and 2022)<br>The code that supports the simulation related analysis of this study is available in https://doi.org/10.5281/zenodo.10728688 .

For manuscripts utilizing custom algorithms or software that are central to the research but not yet described in published literature, software must be made available to editors and reviewers. We strongly encourage code deposition in a community repository (e.g. GitHub). See the Nature Portfolio guidelines for submitting code & software for further information.

Data

Policy information about availability of data

All manuscripts must include a data availability statement. This statement should provide the following information, where applicable:

- Accession codes, unique identifiers, or web links for publicly available datasets
- A description of any restrictions on data availability
- For clinical datasets or third party data, please ensure that the statement adheres to our policy

The datasets generated during and analysed during the current study are available in the [Research Data] repository on heiDATA /Göpfrich Group - Biophysical Engineering of Life with the identifier https://doi.org/10.11588/data/ADYUNN.
Source data are provided with this paper.

Research involving human participants, their data, or biological material

Policy information about studies with human participants or human data. See also policy information about sex, gender (identity/presentation), and sexual orientation and race, ethnicity and racism.

Reporting on sex and gender	n.a.
Reporting on race, ethnicity, or other socially relevant groupings	n.a.
Population characteristics	n.a.
Recruitment	n.a.
Ethics oversight	n.a.

Note that full information on the approval of the study protocol must also be provided in the manuscript.

Field-specific reporting

Please select the one below that is the best fit for your research. If you are not sure, read the appropriate sections before making your selection.

☒ Life sciences ☐ Behavioural & social sciences ☐ Ecological, evolutionary & environmental sciences

For a reference copy of the document with all sections, see nature.com/documents/nr-reporting-summary-flat.pdf

Life sciences study design

All studies must disclose on these points even when the disclosure is negative.

Sample size	No sample-size calculations were performed. Sample size was determined to be adequate based on the magnitude and consistency of measurable differences between groups. This means in detail that the number of analyzed DNA nanotube rings has been estimated to be larger than a group that represents several subgroups of specific features and smaller than limited by the experimental techniques applied.
Data exclusions	No data was excluded from the analysis except for rings with a diameter of less than 1 µm (confocal microscopy). This was necessary to avoid bias due to low resolution.
Replication	Experiments in Figure 1, 2, 3, 4e, 6 have been sucessfully repeated at least twice but reported only once. Simulation experiments have been repeated once and can be replicated with the given code.
Randomization	The imaged sample areas were chosen randomly throughout the study. The experimental group of DNA nanotube rings was chosen to show no side effects of sample preparation but preserving the characteristics of ring-like structures that were under observation. The selection of what is considered as an analyzable ring and thereby distinguished from not investigated sampe is shown in Figure 3.
Blinding	Investigators were not blinded because no group allocation took place.

Reporting for specific materials, systems and methods

We require information from authors about some types of materials, experimental systems and methods used in many studies. Here, indicate whether each material, system or method listed is relevant to your study. If you are not sure if a list item applies to your research, read the appropriate section before selecting a response.

Materials & experimental systems

n/a	Involved in the study
☒	☐ Antibodies
☒	☐ Eukaryotic cell lines
☒	☐ Palaeontology and archaeology
☒	☐ Animals and other organisms
☒	☐ Clinical data
☒	☐ Dual use research of concern
☒	☐ Plants

Methods

n/a	Involved in the study
☒	☐ ChIP-seq
☒	☐ Flow cytometry
☒	☐ MRI-based neuroimaging

Plants

Seed stocks	n.a.
Novel plant genotypes	n.a.
Authentication	n.a.
